# The role of tryptophan in *Chlamydia trachomatis* persistence

**DOI:** 10.3389/fcimb.2022.931653

**Published:** 2022-08-02

**Authors:** Li Wang, YingLan Hou, HongXia Yuan, Hongliang Chen

**Affiliations:** ^1^ The First School of Clinical Medicine, Chenzhou No.1 People’s Hospital, Southern Medical University, Guangzhou, China; ^2^ Department of Clinical Microbiology Laboratory, Chenzhou No.1 People’s Hospital, Chenzhou, China

**Keywords:** C*hlamydia trachomatis*, tryptophan, persistent infection, interferon gamma, indoleamine-2,3-dioxygenase

## Abstract

*Chlamydia trachomatis* (*C. trachomatis*) is the most common etiological agent of bacterial sexually transmitted infections (STIs) and a worldwide public health issue. The natural course with *C. trachomatis* infection varies widely between individuals. Some infections clear spontaneously, others can last for several months or some individuals can become reinfected, leading to severe pathological damage. Importantly, the underlying mechanisms of *C. trachomatis* infection are not fully understood. *C. trachomatis* has the ability to adapt to immune response and persist within host epithelial cells. Indoleamine-2,3-dioxygenase (IDO) induced by interferon-gamma (IFN-γ) degrades the intracellular tryptophan pool, to which *C. trachomatis* can respond by converting to a non-replicating but viable state. *C. trachomatis* expresses and encodes for the tryptophan synthase (TS) genes (*trpA* and *trpB*) and tryptophan repressor gene (*trpR*). Multiple genes interact to regulate tryptophan synthesis from exogenous indole, and persistent *C. trachomatis* can recover its infectivity by converting indole into tryptophan. In this review, we discuss the characteristics of chlamydial infections, biosynthesis and regulation of tryptophan, the relationship between tryptophan and *C. trachomatis*, and finally, the links between the tryptophan/IFN-γ axis and *C. trachomatis* persistence.

## Introduction

*Chlamydia trachomatis* (*C. trachomatis*) is an aerobic, gram-negative, obligate intracellular bacterial pathogen (Chumduri et al., 2013). The World Health Organization (WHO) estimated that there were 131 million newly infected cases of *C. trachomatis* infections among adolescents and adults aged 15-49 in 2012, with a global incidence of 38 infections per 1,000 women and 33 infections per 1,000 men (O'Neill et al., 2018). The estimated number of chlamydial infection cases is most likely to be an underestimation of the actual number of cases, because most natural courses are asymptomatic and go undetected. Up to 70–80% of the *C. trachomatis* infections in women are asymptomatic, and therefore unrecognized and untreated. Studies on the natural course of untreated *C. trachomatis* lower genital tract infections in women show spontaneous clearance rates of 30% in the first weeks or months, approximately 50% in 1 year, 80% in 2 years, and 94% in 4 years (den Hartog et al., 2006; Ouellette et al., 2021).

*C. trachomatis* invades the epithelial cells of the endocervix and upper genital tract in women, as well as the conjunctiva, urethra, and rectum in both men and women (Morrison et al., 2020). In females, the cervix is the most commonly infected site and *C. trachomatis* can enter the uterus and fallopian tubes from the cervix to provoke an inflammatory condition and serious reproductive complications. While the spontaneous clearance of chlamydial lower genital tract infections may lead to an underestimation of the percentage of women at risk of complications, because clearance from the lower genital tract does not mean that the causative agent has not already ascended to the upper genital tract. Most infected women seem to have an adequate immune response, but some infected women will have a long-lasting *C. trachomatis* infection and, subsequently, an increased risk of sequelae, including the development of pelvic inflammatory disease, tubal scarring, ectopic pregnancy, and infertility (Hua et al., 2015).

*C. trachomatis* has the ability to adapt to immune response and persists within host epithelial cells, resulting in an irreversible inflammatory damage. A previous study on asymptomatic non-pregnant women showed the resolution rate of *C. trachomatis* to be 11–45% and could not be treated in a timely manner (Juliana et al., 2021). Untreated women are more likely to get cervicitis, urethritis, pelvic inflammatory diseases, chronic pelvic pain, ectopic pregnancy, miscarriage, and infertility (Yeow et al., 2016; Casillas-Vega et al., 2017). Progression to these sequelae is thought to be the outcome of pathological inflammatory responses such as tissue disruption, fibrosis, and scarring. Moreover, *C. trachomatis* infection may increase the probability of co-infections with human immunodeficiency virus and Neisseria gonorrhoeae. Further, *C. trachomatis* impedes human papilloma virus (HPV)-induced mechanisms that maintain cellular and genomic integrity and it may be linked to cervical cancer (Koster et al., 2022). *C. trachomatis* has also been identified in the tissues of 70% of ovarian tumors and none in benign or normal ovaries, suggesting a potential role in ovarian carcinogenesis (Jonsson et al., 2018). *C. trachomatis* infection is also associated with adverse outcomes in pregnancy, including chorioamnionitis, preterm delivery, and low birth weight (Tamarelle et al., 2019). Nearly half of babies born vaginally to mothers infected with genital *C. trachomatis* will get chlamydial conjunctivitis, some babies will also acquire nasopharyngeal infections and even develop chlamydial pneumonia (Redgrove and McLaughlin, 2014). Revealing that the pathogenic mechanisms of persistent *C. trachomatis* infection is of the utmost importance for developing effective strategies to prevent and control complications.

The genomes of chlamydial strains are remarkably conserved in genetic order and content, except the polymorphic regions of the genome called plasticity zones (PZ). *Trp* resides in the PZ and encodes tryptophan synthase (TS) (Caldwell et al., 2003; Carlson et al., 2004). TS is a heterotetramer composed of two α subunits (TrpA) and two β subunits (TrpB), which catalyzes the two final biosynthetic reactions of tryptophan (Miles, 2001). A distinct difference between urogenital and ocular isolates of *C. trachomatis* is that only the former expresses TS and is therefore able to synthesize tryptophan *via* indole salvage (Carlson et al., 2004; Carlson et al., 2005). Interferon-gamma (IFN-γ) is considered to be a major anti-chlamydial effector cytokine. IFN-γ acts indirectly on cells to limit the tryptophan required for *C. trachomatis* by activating indoleamine-2,3-dioxygenase (IDO) to deplete the intracellular tryptophan pool, resulting in restricted chlamydial growth and development in human epithelial cells. *C. trachomatis* can recover from a persistent state into an infectious state by converting indole into tryptophan *via* TS (Aiyar et al., 2014). We speculate that the synthesis of tryptophan using exogenous indoles in an IFN-γ-rich environment allows the bacteria to escape IFN-γ-mediated immune responses and establish a persistent infection. If true, this would be an important virulence factor and provide a potential mechanism for *C. trachomatis* to evade host defenses. In this review, we summarize the current understanding of changes in molecular and morphological characteristics of *C. trachomatis* during tryptophan starvation, the relationship between tryptophan and *C. trachomatis*, and the influence of the tryptophan/IFN-γ axis on *C. trachomatis* persistence.

### Biphasic life cycle of *C. trachomatis*

Based on the characterization of the major outer membrane protein (MOMP), *C. trachomatis* can be divided into several serogroups, including A, B, Ba, C, D, Da, E, F, G, Ga, H, I, Ia, J, K, L1, L2, L2a, and L3. *C. trachomatis* serovars A–C can cause trachoma that is the dominant cause of blindness, while serovars D–K make Chlamydia the most prevalent bacterial sexually transmitted infection worldwide. Lymphogranuloma venereum (LGV) serovars L1–L3 cause STIs with ulcers and vesicle formation (Bayramova et al., 2018; Murray and McKay, 2021; Sixt, 2021). Genotypes D, E, and F are more prevalent in urogenital infections and are common among heterosexuals. Genotypes D, G, and J are commonly detected in the anorectal tract in women and men who have sex with men (MSM) (Bowden et al., 2021).

*C. trachomatis* demonstrates a unique biphasic life cycle with two morphologically distinct forms: metabolically inactive elementary bodies (EBs) infect host cells through phagocytosis or endocytosis and differentiate into metabolically active replicating reticulate bodies (RBs). EBs differentiate into RBs and begin to replicate within a membrane-bound compartment of the eukaryotic cell in a process known as inclusion (Howe et al., 2019). RBs divide by binary fission, and consequently grow until the entire cytoplasmic matrix is filled and the nucleus is dislocated. RBs adapt in various ways to promote survival and replication: 1) through selective fusion of vesicles to inhibit lysosomal fusion and promote nutrient-rich extracellular vesicles; 2) through a complex isolation mechanism to acquire nutrients such as phosphatidylcholine and cholesterol; 3) through regulation of the host cells intrinsic immunity and cellular survival, among other mechanisms. After 24–74 hours, RBs re-differentiate into EBs, which are then released to initiate a new infection cycle by the lysis of host cell and/or the extrusion of inclusion (Murray and McKay, 2021).

**Figure 1:**
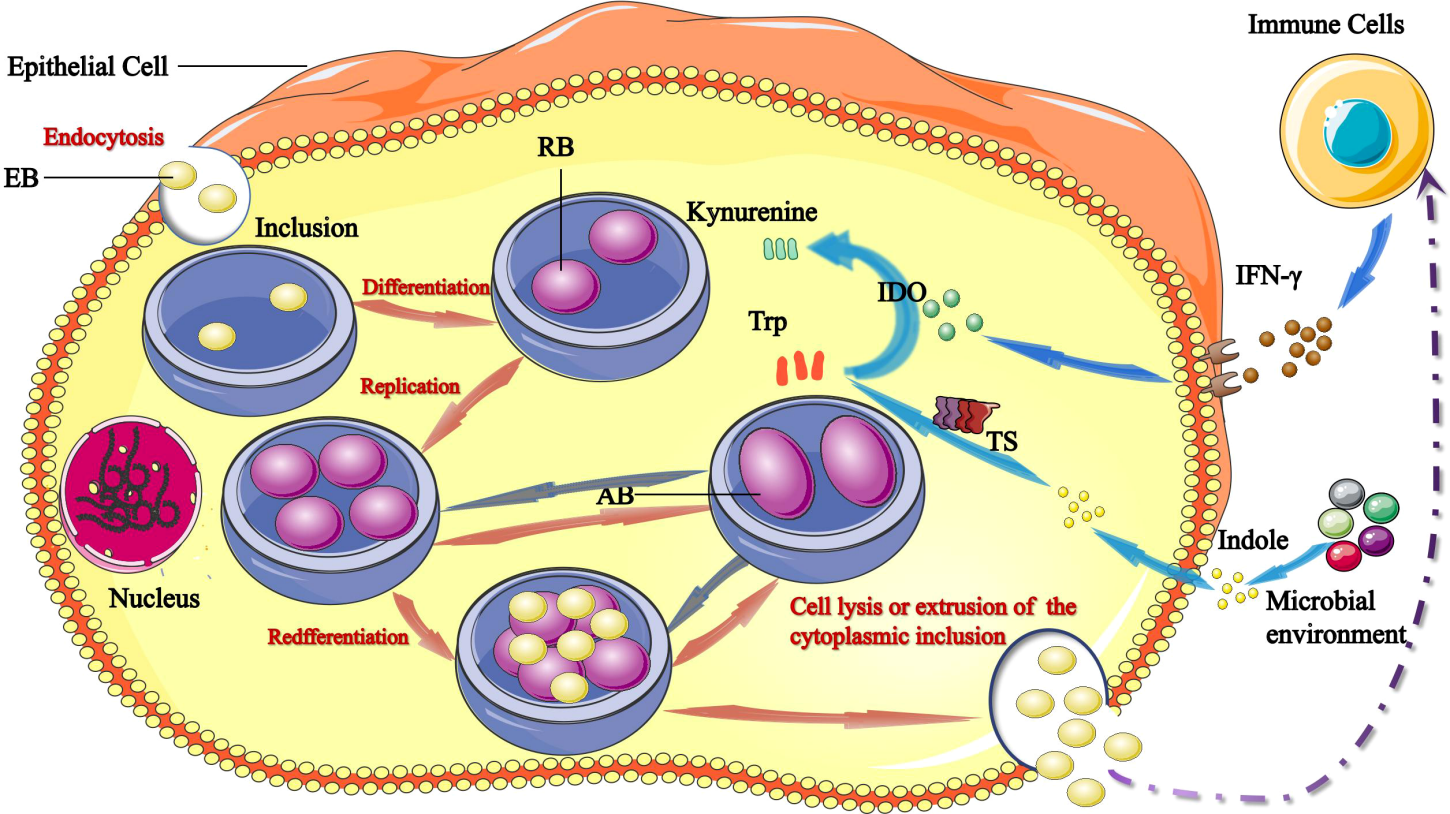
Links between tryptophan/IFN-γ axis and *C. trachomatis*. The infectious form of *C. trachomatis*, elementary bodies (EBs) infect epithelial cells *via* endocytosis. Upon entry, EBs differentiate into reticulate bodies (RBs) and start replicating within the inclusion. RBs divide by binary fission, and consequently grow until the entire cytoplasmic matrix is filled and the nucleus is dislocated. After 24–74 hours, RBs redifferentiate into EBs, EBs are released by host cell lysis and/or the extrusion of inclusion to initiate a new infection cycle. IFN-γ induces production of IDO1, which catabolizes tryptophan into kynurenine. Tryptophan starvation can lead to RBs turn into aberrant bodies (ABs). The chlamydial tryptophan synthase (TS) gene converts indoles produced by other microbiota back into tryptophan. Persistent *C. trachomatis* can become infectious and re-enter the life cycle by converting indole into tryptophan *via* TS.

Another pivotal feature of *C. trachomatis* is its ability to establish persistence, which is a reversible state that occurs in adverse growth conditions. In this state, RBs stop dividing and they turn into aberrant bodies (ABs), which are obviously larger in size and in this form successfully survive. As a persistent infection, *C. trachomatis* re-enters the cycle of development when the host’s immune function is restored, drugs are discontinued and the living environment is improved (Chumduri et al., 2013; Bayramova et al., 2018) (**Figure 1**).

### *C. trachomatis* persistence

Plenty of factors may contribute to persistent infection, including use of antibiotics, nutritional deficiency, cytokine release, viral infection, phages, other diseases, and individual differences (Bayramova et al., 2018). The course of a *C. trachomatis* infection (i.e., whether the infection will be cleared or persistent) may be determined by pathogenic virulence factors, environmental factors, and/or host immune factors. During treatment, about 10–15% of patients are reinfected leading to development of a persistent infection. *C. trachomatis* can establish asymptomatic, persistent infections by several mechanisms, including antibiotic resistance, immune evasion, and suppression of apoptosis (Chumduri et al., 2013). Chlamydial persistence represents a means by which the pathogen resists a key immune response, allowing expression of gene products but inhibiting gene products necessary for rapid pathogen proliferation. Persistent *C. trachomatis* infection is a reversible state under detrimental growth conditions, which is viable but metabolically quiescent, with an enlarged aberrant form. This is a special state that bacteria enter when they are challenged and can regain their pathogenicity when conditions are suitable for survival.

In the host immune system, the most well-recognized biological characteristic of *C. trachomatis* is its ability to stay in contact with the host for a long period of time, during which the bacteria are apparently quiescent or latent. However, this characteristic is also the least understood and is a matter of controversy in the field. The first known article on human chlamydial infection was written by Ritter in 1879 (Radomski et al., 2016). *C. trachomatis* can remain quiescent in a host for a long time and subsequently contribute to a chronic clinical disease. This was first recognized in infections of *Chlamydia psittaci (C. psittaci)*. Between 1879 and 1928, outbreaks of *C. psittaci* that were related to the import of parrots occurred in the United States, Germany, England, France, and Switzerland. People who had close contact with birds would acquire infection from those sick birds. Moulder suggested that this was a “persistent” infection and presumed that it had entered a nonreplicating stage that he termed a “cryptic body”. Unfortunately, he never went on to study these observations further (Rank and Yeruva, 2014).

There has been more evidence of persistent chlamydial infection in the reproductive tract since persistent infection was first defined in 1980, but there is no uniform definition of persistent infection until now. Molecular biology studies suggest that persistent infection is the presence of abnormal body formation in the body, while pathogenesis studies suggest that it is a live but uncultured infection. From a clinical perspective, persistent infection is defined as an asymptomatic infection. The incidence of persistent recurrent chlamydial infection cannot be fully estimated because live chlamydia are difficult to culture (Moulder et al., 1980). *C. trachomatis* has successfully evolved mechanisms to avoid death by autophagy and the host immune mechanisms to persist within host epithelial cells. In persistent chlamydia infection, biosynthesis of membrane and structural proteins, as well as lipopolysaccharides (LPS) stops, while stress-induced production of chlamydial 60-kDa heat shock protein (Hsp60) increases. In addition, synthesis of MOMP, which is a potentially protective antigen, decreases in persistent infections, while levels of the 57-kDa chlamydial heat shock protein (Hsp57) increase, which may contribute to the pathogenesis of the disease. The expression of chlamydial abnormal bodies includes outer membrane proteins, lower levels of LPS, and Hsp60. Hsp60 stimulates production of inflammatory cytokines and the oxidation of low-density lipoproteins (LDLs). Anti-Hsp60 immune reactivities are associated with the pathological consequence of chlamydial infections (LaVerda et al., 1999; Kinnunen et al., 2001; Witkin et al., 2017). RBs stop dividing, after which they turn into ABs with an obviously larger size and they do not produce a new parent (EB). The AB state is a reversible state that occurs in adverse growth conditions, in which *C. trachomatis* stops replicating and has a reduced capacity to proliferate and metabolize. It has continued chromosomal replication and active biosynthetic processes. RBs enter a persistent non-replicative but viable state under unfavorable conditions. The infectious form of the organism, the EB, is again generated when immune attack subsides (Witkin et al., 2017).

*C. trachomatis* is highly dependent on the biochemical processes and nutrient supply from the host cell. Growing evidence suggests that tryptophan plays a significant role in chlamydial growth and pathogenesis, which has generated interest in the regulatory mechanisms of tryptophan expression. Under conditions of tryptophan deficit, *C. trachomatis* can use its own synthesis to maintain tryptophan metabolism in a stable state, and when host cells are severely deficient in tryptophan, *C. trachomatis* activates self-protective mechanisms and enters a state of persistent infection to survive (Bommana et al., 2021) (**Figure 1**).

### Abnormal development in chlamydial persistent infection

In chlamydial persistent infection, genes including tryptophan synthesis (*trpR, trpB, and trpA*), phospholipid biosynthesis *(lpd, lpxK, lipA and pgsA.2*), DNA repair and recombination (*xerC, dnaB, recA and yqgF*), translation (*trmD, rl21, fusA, rs19, tsf, efp.2, ytgB.2, infC*) and many early cycle genes (*incD-G and euo*) were highly upregulated. *FtsW* (a chaperone of PBP 3) and *AmiA* (an amidase) were significantly down-regulated, suggesting that cell division was blocked in persistent infection. In addition, glyceraldehyde-3-phosphate dehydrogenase (GAPDH) was involved in many crucial pathways in bacteria. The expression of *gapA* (GAPDH) changed in the presence of IFN-γ. GAPDH was an essential enzyme in the glycolytic and pentose phosphate pathway that reduced NAD+ to NADH by oxidizing glyceraldehyde-3-phosphate (GAP) to 1,3-diphosphoglycerate. Previous studies have shown that the expression of chlamydial GAPDH was associated with colonization, pathogenesis, virulence and chlamydial growth. Furthermore, GAPDH functioned in both phases of the biphasic life cycle of *C. trachomatis* (Belland et al., 2003; Barrett et al., 2020; Schormann et al., 2020).

The protein product of euo has been thought to repress late transcription and its expression was significantly up-regulated in persistent *C. trachomatis*, upon removal of IFN-γ, the product was significantly decreased. Nevertheless, increased expression of euo was currently thought to be correlated with glucose deprivation, but not persistence (Belland et al., 2003).

Furthermore, *pH2AX, H3K9me3, gH2AX* induced by DNA double-strand breaks (DSBs) and senescence-associated heterochromatin foci (SAHF) showed upregulation during infection. Dysregulation of host cell signal, disrupting host chromatin, repair of DSBs and regulation of cell cycle were likely to form malignant transformation (Chumduri et al., 2013).

### Biology of tryptophan

#### The tryptophan synthase operon

*C. trachomatis* has developed a mechanism to evade host immune defenses by retaining a subset of *trp* genes, encoded in the PZ: *trpA* and *trpB*, encoding homologs of the α (TrpA) and β (TrpB) subunits of TS; and *trpR*, encoding a tryptophan repressor (Carlson et al., 2006; Aiyar et al., 2014; Ziklo et al., 2016a). *TrpRBA* are located in the PZ of the *C. trachomatis* genome, which is approximately 50 kb in length and accounts for most of the sequence variations in *C. trachomatis* strains (O'Neill et al., 2018). TrpAB is a bifunctional enzyme catalyzing the final two steps. The enzyme is a heterotetramer consisting of two α subunits and two β subunits (αββα) (McClarty et al., 2007). Upregulation of the *trpBA* operon converts indole to tryptophan. *TrpR* binds an operator sequence (*trpO*) and inhibits *trpBA* transcription. When sufficient tryptophan is present, *trpR* inhibits transcription of the tryptophan operon and transcription of other additional genes, including *trpR* itself, *aroH*, *aroL*, and *mtr*. In the presence of low tryptophan, *trpR* is released from the *trpO* to stimulate gene expression (Wood et al., 2003; Akers and Tan, 2006; Banerjee and Nelson, 2019).

In *C. trachomatis*, catalytically impaired TrpA is an allosteric regulator of TrpB. TrpA can improve TrpB activity by four-fold. TrpAB activity is influenced to promote the open state by monovalent cations, such as NH_4_^+^, Cs^+^, K^+^, and Na^+^ (Michalska et al., 2021).

TrpA (subunit α) is a TIM-barrel, whereas TrpB (subunit β) consists of two 3-layer structural domains (αβα). Further, TrpA catalyzes the α reaction, converting indole-3-glycerol phosphate into indole and glyceraldehyde-3-phosphate. Indole is subsequently utilized by TrpB. TrpB catalyzes the β-replacement reaction, in which indole takes the place of the hydroxyl radical of serine into tryptophan. Within each αβ heterodimer, subunit α and subunit β act independently but in a highly coordinated fashion. Both subunits perform two functions: allosteric communication and catalysis (Dunn, 2012; Michalska et al., 2021).

Sherchand et al. have demonstrated that the indole metabolites, indole-3-propionic acid (IPA) and indole-β-acrylic acid (IAA), compete with tryptophan for binding to TrpR to inhibit its binding to TrpO. IPA and IAA are deleterious to urogenital *C. trachomatis* through de-repressing *trpRBA* Expression and indole-producing microbiomes prevent chlamydial immune clearance by TS. The presence of indole allows tryptophan biosynthesis to evade starvation and blocks serine deamination (Sherchand and Aiyar, 2019).

#### Tryptophan synthesis

Tryptophan is an essential aromatic amino acid in the growth and development of *C. trachomatis*. Among the 20 common essential amino acids, tryptophan is the least abundant by protein content and the largest by molecular weight. Tryptophan is unique in that it is specified by just one codon. The amount of tryptophan is generally low, which is biochemically the most expensive to synthesize *in vivo* (Lo et al., 2012). Some organisms can synthesize tryptophan *de novo via* the successive action of a series of enzymes (Wood et al., 2003). The entire tryptophan synthetic pathway requires the sequential action of seven enzymes (TrpA-TrpG) and 78 ATP molecules. Given the high metabolic burden, the pathway is strictly controlled in multiple aspects (Michalska et al., 2021). Comparative genomics of *C. pecorum* has revealed that *C. pecorum* possesses a near complete tryptophan operon (*trpRDCAB*), and *C. pecorum* isolates can escape IFN-γ effects by depleting tryptophan expression in human epithelial cells (Islam et al., 2018). Most of the sequenced chlamydial genomes do not contain the full complement of genes required to synthesize tryptophan, six (*trpA, B, C, D, F, R*) in *C. psittaci*, four (*trpA, B, F, R*) in *C. trachomatis* serovar D, one (*trpF*) in *C. muridarum*, and none in *C. pneumoniae*. Different susceptibilities of chlamydial strains to IFN-γ may be due to variations in tryptophan synthesis. As an infant passes through the obstetric canal, genital *C. trachomatis* can also infect the conjunctivae, which leads to a self-limiting conjunctivitis rather than trachoma (Caldwell et al., 2003). This suggests that genital strains are highly sensitive to the inhibitory effects of IFN-γ in the ocular environment, perhaps because there is no exogenous indole.

#### Tryptophan metabolism

Tryptophan metabolism follows three major pathways: 1) tryptophan is directly converted into several molecules, including the ligands for the aryl hydrocarbon receptor (AhR); 2) tryptophan is transformed into kynurenine (kynurenine pathway (KP)) *via* IDO in both immune and epithelial cells; and 3) tryptophan is metabolized to the serotonin (5-hydroxytryptamine (5-HT)) production pathway *via* tryptophan hydroxylase 1 (TpH1) in enterochromaffin cells (Agus et al., 2018). Metabolic tryptophan pathways, including the 5-HT, KP, and AhR pathways, play an important role in physiology and pathophysiology.

### Tryptophan starvation contributes to *C. trachomatis* persistence

*C. trachomatis* regulates the tryptophan biosynthetic pathway by encoding *trp* genes. When the host limits the bioavailability of tryptophan through defense mechanisms, *C. trachomatis* can use this mechanism to maintain its own tryptophan metabolism at a steady state, and when host cells are severely deficient in tryptophan, *C. trachomatis* turns on self-protective mechanisms to maintain its integrity and survival by entering a state of persistent infection. The most prominent feature of the AB phenotype is an increase of TrpA and TrpB levels from under 0.05% of total protein in EBs to 9% in ABs. The differentiation process of EBs to RBs, and RBs to ABs is highly dependent on tryptophan, indicating that TrpAB may have an advantage in infection. Furthermore, the total tryptophan content in the AB form is 1.9-fold lower compared to the EB form (Ostergaard et al., 2016).

Jordan et al. have demonstrated significantly lower tryptophan levels and a trend towards lower IFN-γ levels in women who cleared their infection compared to others with persistent infection. High levels of cervicovaginal tryptophan were detected in most women who had spontaneously cleared their chlamydial infection (Jordan et al., 2017).

*C. trachomatis* is auxotrophic for tryptophan and must obtain the essential amino acid from the external environment. *C. trachomatis* is an obligate intracellular bacterium which scavenges tryptophan from the host. These bacteria obtain this essential amino acid from host cells through a pool of free amino acids in the cytoplasm or by ingesting enzymatic degradation products. Deprivation of tryptophan represses chlamydial replication and prevents the spread of infection by blocking the differentiation of RBs into EBs, making tryptophan synthesis a pathogenic strategy for evading the host defenses within an IFN-γ-rich environment (Ouellette et al., 2021).

Ibana et al. have demonstrated that IDO1 inhibitor 1-methyl-Trp (L-1-MT) limited the number of EBs, reduced the numbers of inclusions, blocked IFN-γ-mediated persistence of *C. trachomatis*, enhanced efficacy of chlamydial antibiotic clearance and increased the susceptibility of persistent infection in response to doxycycline treatment. Adding L-1-MT to reactivate the persistent *C. trachomatis* induced by IFN-γ restricted the productive multiplication of the bacterium, which might be due to the inhibition of the enzyme. Furthermore, the available tryptophan was insufficient to support normal late-stage development of bacteria (Ibana et al., 2011).

Recently, there has been increased interest into the correlation between genes encoding tryptophan codons and persistence. Our previous work and that of others have demonstrated that *C. muridarum* colonizes the genital tract and establishes long-lasting colonization in the intestinal tract (Hongliang et al., 2022). Urogenital *C. trachomatis* strains can utilize exogenous indole to synthesize tryptophan using *trpBA*. Several genes interact to regulate the tryptophan synthesis from exogenous indoles, and *C. trachomatis* can recover its infectivity from persistence by converting indole into tryptophan. If tryptophan is added, ABs can be reactivated and enter a normal growth cycle (Ostergaard et al., 2016; Michalska et al., 2021). Depletion of tryptophan may lead to inhibition of T cell proliferation and increased cell apoptosis (Chen, 2011).

### Iron-dependent repressor YtgR regulates the gene expression of trp

The *trp* operon contains *trpR*, *trpB* and *trpA* and an intergenic region (IGR). *TrpR* regulates the major promoter (*PtrpR*), while the IGR has the iron-dependent repressor YtgR and a promoter (*PtrpBA*) to regulate *trpBA* expression. YtgR is the only known iron-dependent regulator in *C. trachomatis* that binds to the *trpRBA* promoter (P*trpRBA*) and inhibits transcriptional initiation of the *ptrpBA* responding to iron and/or tryptophan starvation. At the same time, binding of YtgR promotes transcriptional termination of the main promoter upstream of *trpR*. Thus, YtgR represents an alternative strategy to attenuate *trpBA* expression. When iron levels are high, YtgR locks on to the DNA in the middle of this set of genes, which effectively shuts down the genes on either side of the binding site. When iron levels drop, YtgR is released from the DNA, allowing renewed tryptophan synthesis (Pokorzynski et al., 2019; Pokorzynski et al., 2020).

The decrease in YtgR levels activates *trpBA* expression from *PtrpBA*, which is regarded as an alternative attenuation strategy in tryptophan regulated prokaryotes. Tryptophan deprivation limits expression of the YtgR repressor to activate transcription from the alternative YtgR-regulated promoter for *trpBA*. This regulatory mechanism parallels cis attenuation by *trpL* in *Escherichia coli (E. coli)*, though notable differences exist: namely, this attenuation mechanism is mediated by a trans-acting transcription factor (Pokorzynski et al., 2020).

### Links between tryptophan/IFN-γ axis and *C. trachomatis* persistence

One of the most important cellular immune responses in the development of protective immunity against chlamydial infection is characterized by antigen-specific IFN-γ- secreting CD4+ and CD8+ T cells. IFN-γ is a critical cytokine in innate and adaptive immune responses against a broad spectrum of bacterial and viral infections, including *C. trachomatis* (Belland et al., 2003; McClarty et al., 2007; Ziklo et al., 2016a). While high IFN-γ levels were shown to eradicate the infection, lower levels can also drive urogenital *C. trachomatis* to enter their persistence form, characterized by aberrant, non-infectious bodies *in vitro* (Ziklo et al., 2018; Ziklo et al., 2019). IFN-γ is a major part of the anti-chlamydial immune response and the most important factor.

One hypothesis suggests that indole-producing bacteria might play an important part in *C. trachomatis* infections, allowing *C. trachomatis* to counteract the IFN-γ mediated immune response by providing indole. *C. trachomatis* bacteria and humans do not produce indole, but microorganisms that colonize the genital and gastrointestinal tract do (Muramatsu et al., 2016). Indole-producing microorganisms such as various anaerobic vaginal floras, including but not limited to, *E. coli*, *Peptostreptococcus* spp., *Fusobacterium* spp., *Bacteroides* spp., are likely to be widespread in the genital and gastrointestinal tracts, which could be a direct source of indole or tryptophan. Interestingly, indole-producing bacteria are more prevalent in women who experience bacterial vaginosis. The incidence of secondary complications of chlamydial infection increases during vaginosis co-infection with *C. trachomatis* (McClarty et al., 2007). Previous studies have shown that indole has been detected in genital secretions from women who were infected with *C. trachomatis*, and women with repeated *C. trachomatis* infections had significantly increased vaginal kynurenine/tryptophan ratios (Ziklo et al., 2018; Ziklo et al., 2019).

Clinical observations suggest that levels of IFN-γ in the infected intracervical microenvironment were higher than normal levels (Ibana et al., 2018). Indole generation at microbial sites allowed tryptophan production by TS, thus avoiding interferon-induced tryptophan starvation by providing exogenous indole (Sherchand and Aiyar, 2019). Lactobacillus was the dominant and protective vaginal microbiota in females, which could kill numerous vaginal pathogens, including *C. trachomatis*. Indole was absent or present in very small amounts in the genital secretions when Lactobacillus was dominant. However, when non-lactobacilli were predominant, such as bacterial vaginosis, indoles were easily detectable (Witkin et al., 2017). Indole was detected in the genital secretions of two infected women with *C. trachomatis* (Ziklo et al., 2018). Under tryptophan-deficient conditions, vaginal specimens with high indole levels had higher recovery of *C. trachomatis*, which provided preliminary support for the crucial effect of the interferon-γ-tryptophan-indole axis. The introduction of exogenous indoles into cell culture media could rescue urogenital *C. trachomatis* strains from IFN-γ and allowed them to subsequently produce infective progenies (Ziklo et al., 2016b).

The anti-chlamydial effects of IFN-γ in humans include the induction of nitric oxide from arginine by nitric oxide synthase, thereby triggering iron deprivation as well as conversion of L- tryptophan into L-formyl kynurenine *via* IDO. L-formyl kynurenine has a wide range of potential roles in the host, such as a precursor to NAD(P) or conversion to acetyl-coenzyme A (acetyl-CoA) in the mitochondria. In addition, IFN-γ strongly induces synthesis of host tryptophanyl-tRNA synthetase, whose elevated level not only favors host access but also tends to sequester the already diminished tryptophan pool away from parasitic metabolism, IFN-γ-induced synthesis of host tryptophan-tRNA synthase not only facilitates host entry but also tends to isolate the already reduced tryptophan pool away from parasitic metabolism (Lo et al., 2012).

IDO is a key protein in human intracellular immune defense against *C. trachomatis*, which limits the growth of the tryptophan auxotroph. Therefore, the enzyme restricts *C. trachomatis* growth. The consequences of tryptophan starvation for *C. trachomatis* are a double-edged sword: the RBs may die leading to clearance of the infection, or the RBs may become persistent (Aiyar et al., 2014; Witkin et al., 2017; Banerjee and Nelson, 2019). *C. trachomatis* may use TS to synthesize tryptophan inside the inclusion, but IDO cannot enter the inclusion (Banerjee and Nelson, 2019).

### The aryl hydrocarbon receptor—The downstream product of tryptophan binds to this receptor

The AhR was first characterized as a protein binder and mediator of the toxic effects of 2,3,7,8-tetrachlorodibenzo-p-dioxin (TCDD, dioxin) and dioxin-like compounds (DLCs). AhR is a cytoplasmic receptor and transcription factor, which is activated primarily by binding to a cognate ligand. AhR is vital for a broad array of immune functions. AhR is also an intracellular receptor of the basic helix-loop-helix (bHLH) gene family. Subsequent studies have demonstrated that the AhR plays a pivotal role in maintaining cellular homeostasis and in pathophysiology. The AhR can bind structurally diverse compounds, which may function as endogenous ligands (Beischlag et al., 2008; Avilla et al., 2020). AhR is thought to be an important molecule in various cellular processes, such as tumorigenesis, embryogenesis, and inflammation. However, the molecular mechanisms of AhR in persistent infections is unknown (Murray et al., 2014).

It is an established hypothesis in the field, *C. trachomatis* infection triggers an IFN-γ response that upregulates IDO1 and IDO1 depletes tryptophan to kynurenine. Kynurenine has been shown to be an important scavenger for free radicals *in vitro*, facilitating the pathogen’s resistance to host-induced oxidative stress; and kynurenine has been demonstrated to affect the morphology of epithelium, which may provide a more suitable environment for chlamydial to infection (Bellmann-Weiler et al., 2010). The establishment of *C. trachomatis* model revealed that IDO-mediated effects not only inhibited the growth of *C. trachomatis*, but might be also involved in the persistence of this pathogen (MacKenzie et al., 2007). Belladonna et al. found that tolerogenic dendritic cells (DCs) could grant suppressive ability in an IDO-dependent fashion to immunogenic DCs, and kynurenine—a downstream metabolic product of tryptophan—can initiate DC-dependent inhibition of T cell growth independent of tryptophan deprivation. (Belladonna et al., 2006).

Kynurenine is an intermediate affinity AhR ligand that has been implicated in regulatory T cell (Treg) functional maturation and suppression of inflammatory cytokine production in dendritic cells. Moreover, several downstream Kyn metabolites such as kynurenic acid, xanthurenic acid, and cinnabarinic acid are potent AhR ligands that have been implicated in modulating cancer cell migration and cytokine production in mouse and human T cells. Tryptophan can also be altered by oxidative reactions to the AhR ligands 2-(1’H-indole-30-carbonyl)-thiazole-4-carboxylic acid methyl ester (ITE) and 6- formylindolo[3,2-b] carbazole (FICZ) in mouse and human cells (Shinde and McGaha, 2018; Platten et al., 2019).

**Figure 2:**
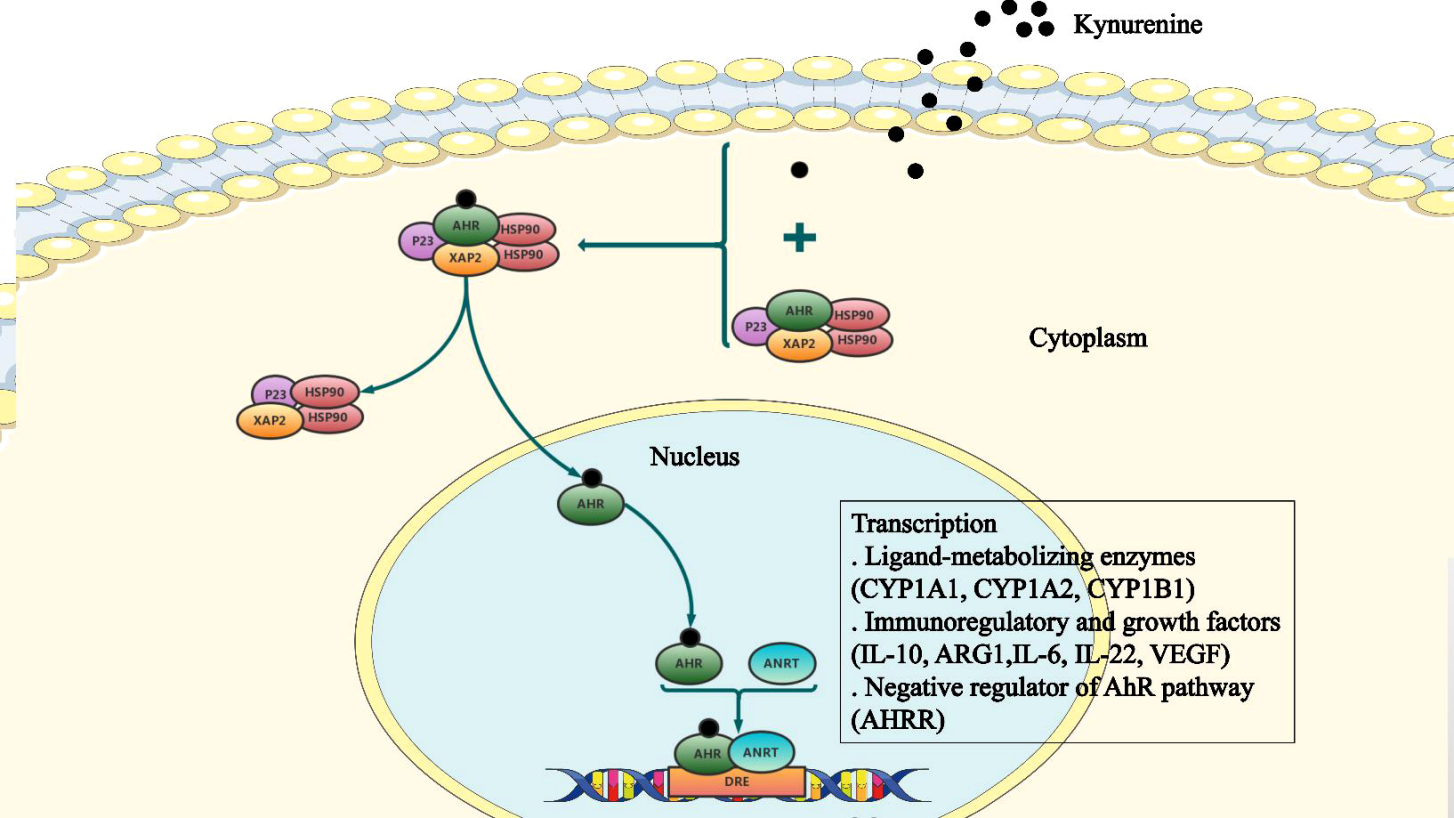
The cellular AhR signaling pathway. The aryl hydrocarbon receptor (AhR) binds to a complex of Hsp90, X-associated protein-2 (XAP2), and P23, which is present in the cytoplasm in a dormant state. Following ligand binding, AhR undergoes conformation changes, the nuclear localization signal (NLS) is exposed, and Hsp90 is released from the complex. Subsequently, AhR forms a heterodimer with the AhR nuclear translocator (ARNT). This heterodimer binds to the xenobiotic response element (XRE) to alter expression of downstream target genes.

Once bound to the ligand, AhR is activated by a conformational change exposing the nuclear localization signal (NLS), which acts as a transcription factor that affects gene expression *via* recruitment of coactivators and corepressors of targeted DNA regions, promoter binding, and cellular signal transduction mechanisms (Sun et al., 2020). AhR functions by interacting with various ligands, including PAS heterodimerization partners AhR nuclear translocator (ARNT), chaperone proteins, and immunophilin-like proteins, including heat shock protein 90 (Hsp90) and AhR-Interacting Protein p23. The complex releases Hsp90 allowing the receptor to translocate to the nucleus. Subsequently, activated AhR and ARNT form a heterodimer, which binds to the xenobiotic response element (XRE) leading to transcriptional changes in ligand-metabolizing enzymes (CYP1A1, CYP1A2, CYP1B1), immunoregulatory and growth factors (IL-10, ARG1, IL-6, IL-22, VEGF), as well as the negative regulator of the AhR pathway (AHRR) (Avilla et al., 2020; Safe et al., 2020; Sun et al., 2020) (**Figure 2**).

AhR is a key factor in limiting macrophage mediated inflammation and may play a dominant role in macrophage polarization and induction of suppressive immune responses. AhR also controls the differentiation of dendritic cells from monocyte precursors, which function as a vital regulator of inflammatory potential for dendritic cells. Moreover, IFN-γ increases the expression and activity of IDO, and in macrophages and dendritic cells may increase kynurenine synthesis, which induces TGF-β1 production and limits the inflammatory response through a feedback mechanism (Shinde and McGaha, 2018).

### The tryptophan metabolic pathway promotes *C. trachomatis* persistence *via* modulation of host immune responses

Chlamydial infection provokes an IFN-γ response resulting in IDO production. Importantly, IDO inhibits chlamydial growth by converting tryptophan to kynurenine to deplete host tryptophan pools. IFN-γ is also thought to act indirectly on *C. trachomatis* cells to limit the tryptophan required for *C. trachomatis* by activating IDO (Lo et al., 2012; Murray and McKay, 2021; Ouellette et al., 2021). Genital *C. trachomatis* strains can overcome this tryptophan issue by encoding an enzyme for tryptophan synthesis. Leonhardt *et al.* have found that a proportion of bacteria are unable to enter the persistent state at very low tryptophan levels (Leonhardt et al., 2007).

Tryptophan is essential for T cell growth and proliferation. A metabolic pathway can fine tune host mucosal responses according to tryptophan catabolism to maintain the evolution of immune physiology, through regulation of AhR-mediated transcription of IL-22. AhR facilitates immune homeostasis, which plays an antibacterial role by regulating AhR-mediated transcription of IL-22, and it also confers anti-inflammatory effects by mediating Treg differentiation (Zelante et al., 2013).

Tryptophan depletion can cause T cells to arrest in the G1 phase of the cell cycle leading to reduced proliferation. Moreover, tryptophan catabolites can induce T cell apoptosis. Kynurenine itself and kynurenine derivatives can stimulate primary naive T cells to differentiate to Tregs and promote subsequent development of Tregs *via* the AhR pathway. IDO can function as a signal transducer to promote the tolerogenic phenotype in plasmacytoid dendritic cells (pDCs) and pDC-induced forkhead box P3 (FoxP3) in the presence of transforming growth factor β (TGF-β) (Chen, 2011; Ziklo et al., 2019).

In addition, products of IDO can induce FoxP3 expression (an essential transcription factor for Treg immunosuppression), promote Treg synthesis, block Th17 cell proliferation and regulation of Th17 cell differentiation. Th17 cells can synthesize IL-17 and they are implicated in host defenses as well as chronic inflammatory diseases. There is also interesting data demonstrating an association between IDO enzyme activity, Th17 differentiation, and Treg from primary naive T cells (Favre et al., 2010).

## Conclusions

In this review, we have described the relationship between tryptophan and *C. trachomatis*, as well as the mechanism by which IFN-γ functions as a protective cytokine against chlamydial infections. We have also discussed the role of IFN-γ-mediated chlamydial virulence responses, and the ways in which different genetic host predispositions vary between host cell types. The protective effect of IFN-γ is due to catabolism of tryptophan by IDO. Tryptophan starvation can stimulate a viable but non-culturable persistent phenotype in *C. trachomatis* that can be reactivated when the IFN-γ response is diminished and/or tryptophan is available. Therefore, overcoming tryptophan starvation may provide a mechanism by which *C. trachomatis* can cause persistent and chronic infection, despite the presence of IFN-γ. Genomic analyses suggest that genital *C. trachomatis* can convert indoles into tryptophan to maintain selective pressure. In contrast, ocular isolates cannot synthesize tryptophan. Given that the *trpBA* gene is present in the PZ, indole availability may be a selective factor to allow indole salvage.

Faced with similar IFN-γ stress as genital strains, it is important to understand why ocular strains have lost TS activity and what other biological differences may exist that may contribute to tissue tropism and disease pathogenesis. In the meantime, further studies are needed to characterize the microbial environment of the reproductive and gastrointestinal tract during chlamydial infection to assess the full impact of the TS gene on chlamydial diseases pathogenesis.

A more comprehensive understanding of the role of tryptophan in mediating *C. trachomatis* infection and pathogenesis and the differences among women in factors affecting clinical outcomes is an important goal of future research. Using such knowledge will aid the development of more personalized and targeted treatments to reduce incidence of adverse outcomes.

## Author contributions

Conceptualization: LW and HC. Writing—original draft preparation: LW. Writing—review and editing: LW, YH, HY and HC. funding acquisition: HC. All authors have read and agreed to the published version of the manuscript.

## Funding

This work was funded by Hunan Provincial Natural Science Foundation of China (2021JJ70002) and Chenzhou Science and technology Bureau (zdyf 2020035 and yfzx201908).

## Conflict of interest

The authors declare that the research was conducted in the absence of any commercial or financial relationships that could be construed as a potential conflict of interest.

## Publisher’s note

All claims expressed in this article are solely those of the authors and do not necessarily represent those of their affiliated organizations, or those of the publisher, the editors and the reviewers. Any product that may be evaluated in this article, or claim that may be made by its manufacturer, is not guaranteed or endorsed by the publisher.
